# The Impact of Covid-19 Pandemic Lockdown During Spring 2020 on Personal Trainers' Working and Living Conditions

**DOI:** 10.3389/fspor.2020.589702

**Published:** 2020-12-10

**Authors:** Solfrid Bratland-Sanda, Therese Fostervold Mathisen, Christine Sundgot-Borgen, Jorunn Sundgot-Borgen, Jan Ove Tangen

**Affiliations:** ^1^Department of Sport, Physical Education and Outdoor Studies, University of South-Eastern Norway, Bø, Norway; ^2^Faculty of Health and Welfare, Østfold University College, Fredrikstad, Norway; ^3^Department of Sports Medicine, Norwegian School of Sport Sciences, Oslo, Norway

**Keywords:** mental health, wellbeing, occupational health, physical activity, public health, exercise, fitness

## Abstract

**Purpose:** The aim of this study was to map changes in working and living conditions of Norwegian personal trainers (PTs) during the Covid-19 lockdown spring 2020.

**Methods:** A total of 150 PTs (mean (SD) age 35.5 (8.4) years, with 6.5 (5.3) years of experience as PT, and 61% female) in Norway responded to an online survey.

**Results:** Number of PT sessions per week was reduced during the lockdown and the majority of the respondents reported loss of clients and negative impact on their working conditions. The official restrictions and guidelines were considered difficult to interpret and somewhat discriminatory compared to restrictions and guidelines for other comparable exercise occupational groups. The lockdown period provided more PTs to offer online and outdoor training. Living conditions were also affected with reported impairments in private economy and reduced vitality (*p* < 0.05), and with vitality being lower in female compared to male respondents during, but not before, the lockdown period.

**Discussion:** The reported negative impact of the Covid-19 lockdown period on PTs' working and living conditions are discussed with focus on the reported gender differences and considering the previously reported poor reputation of the fitness industry.

## Introduction

Severe acute respiratory syndrome coronavirus 2 (SARS-CoV-2) causes an infectious disease (Covid-19) and has per July 2020 spread to more than 200 countries and infected more than 15 million people (WHO, 2020). SARS-CoV-2 is a highly transmissible virus that is primarily spread through airborne droplets when infected individuals congregate with others in enclosed spaces (Morawska and Milton, 2020). The outbreak of the Covid-19 pandemic in Norway led to a national lockdown starting on March 12th, 2020. The lockdown included among others closing of all fitness centers, organized sports and sports events, and the whole community was advised to keep a social distance of two meters (The Norwegian Directorate of Health, 2020). Such intervening specifically affected those employed within the private and/or civil sector (e.g., freelancers, self-employed persons, part-time staff within culture, sport and fitness), and many suddenly found themselves without jobs and reduced income. The first 2 weeks of lockdown were followed by a gradual re-opening of the community in prioritized order. Operators within the health sector were specifically prioritized, and authorized health personnel such as physiotherapists and wellness actors such as massage therapists could reopen their practices from April 27th 2020. Schools reopened on May 11th 2020, organized youth-sports carefully returned toward normal operation from June 1st 2020; while fitness facilities were hold on wait until June 15^th^ 2020. For the occupational group of personal trainers (PT), no specific recommendations concerning return to work and reopening of practice were made besides the opening of fitness facilities.

The occupational group of personal trainers (PTs) in Norway is an increasing group, estimated to ~3,000 in number, and with official reports of an exponential growth in service sales (Virke Trening, 2019). Yet, there is limited knowledge about this profession and their working conditions. There is no requirement of formal accreditation to work as a PT, and a report from 2016 showed that 84% of the Norwegian PTs lack a formal university degree in exercise science (Virke Trening, 2016). Malek et al. (2002) showed that formal education in exercise science, and not years of work experience, predicted the PTs' knowledge about nutrition, health screening, testing, exercise prescription, and clinical exercise physiology. Norway also lacks a formal accreditation of clinical exercise physiologists such as seen in e.g., Australia (Cheema et al., 2014; Soan et al., 2014; Lederman et al., 2016), and PTs with and without such exercise-specific higher education are thus given the same rights, privileges and responsibilities when performing their profession. Although the fitness industry has been mentioned as an important partner for physical activity, and hence public health, in white papers and global actions for physical activity by the World Health Organization (WHO, 2018) and by the Norwegian government (Departementene, 2020), little has been done to formally develop this partnership by the authorities. This lack of formal partnership might be viewed in light of poor reputation of the fitness center industry in Norway, mainly due to the impression of the industry's focus on cosmetics/aesthetics, body and appearance (Klepaker and Norøy, 2019). The prolonged lockdown of the industry was also maintained despite attempts from the fitness industry trade organization *Virke trening* to develop standards and guidelines for infection control within fitness centers (Kristiansen, 2020a). The industry thus questioned the late opening of the fitness centers, in addition to the lack of any concrete guidelines addressing PTs during the lock-down reopening phase, yet no empirical data exists on how the PTs experienced this lack of concrete guidelines.

Across several occupational groups and sectors, there has been shown important gender differences in e.g., sick leave, occupational health and family obligations. Women show higher sick leave rates compared to men (Barmby et al., 2002), and numbers from Swedish statistics (Angelov et al., 2013) showed that sickness absence in women were between 0.5 and 0.85 days per month higher than in men when entering parenthood. The most plausible explanation for this difference is the inequality in family responsibilities between men and women (Angelov et al., 2013). Studies on other occupations, such as teachers, also report poorer health and well-being, higher job stress and lower job satisfaction among females compared to males (Klassen and Chiu, 2010). Due to the aforementioned scarce knowledge about PTs' working status and pattern, potential gender issues and perspectives are important to examine.

The Covid-19 pandemic and actions to reduce infection spreading, such as lockdown, quarantine and isolations, have a huge impact on several levels (Raymond et al., 2020; Shammi et al., 2020; Wang et al., 2020). It affects societies, economies, spread fear, and hence potentially impairs lifestyle behavior and increase mental health challenges (Carbone, 2020). Impacts such as loss of income, the experience of not being evaluated as adequately important to reopen business, and the isolation from social interaction may have profound effects on health (Holmes et al., 2020). Besides grief, stress, anxiety and depression; changes in lifestyle behavior such as sleep, eating and physical activity may also cause somatic health impairment (Woods et al., 2020). Also, it is reasonable to believe that the increase in mental health challenges also can contribute to a rice in suicide rates (Kawohl and Nordt, 2020). It is thus possible that these detrimental consequences of the lockdown also influence the PTs' own health and lifestyle.

The Norwegian government acknowledged the huge impact and potential financial crisis on companies and businesses of the forced lockdown, and thus developed an *ad hoc* financial compensatory arrangement for companies and self-employed who experienced losses of minimum 20% of their income in March 2020 and 30% loss of income in April 2020 (Norwegian Government, 2020). Nevertheless, the arrangement, administered by the Norwegian Labor and Welfare Organization (NAV), has been debated in the media for not being sufficient (Arnesen and Jordal, 2020). The lock-down of fitness centers has led to a documented loss of members terminating their memberships (Revfem, 2020), which may have profound impact on the PTs' personal economy and future income and personal economy.

This descriptive study therefore aims to expose the impact of Covid-19 and the enforced lockdown on the work and living conditions of the PTs, and to explore whether there were gender differences in these conditions. We wanted to detect how many PTs were on lay off temporarily, which changes in working hours and sessions they experienced, and whether they managed to reorganize and offer new products using digital platforms and outdoor facilities. Furthermore, the PTs perspectives on the general restrictions and guidelines for the lockdown and the infection control were of interest. We also wanted to describe if and how the pandemic, as well as the lockdown itself, had an impact on the PTs' routines of exercise, their health, and their view on their future as PT. Finally, we examined the PTs' educational background, their working conditions, and whether they had other/additional occupations in addition to their PT occupation or received economic support from the NAV.

## Materials and Methods

### Design

This is a cross-sectional, national cohort study inviting PTs to respond to a digital, anonymous survey on experiences related to personal health and -economics, and to the communication of- and interpretation of regulations during the Covid-19 lockdown. Written information about the study and its purposes was made available together with a link to the survey in all recruitment areas. The PTs were recruited through announcement in social media (through researchers' personal posting and sharing on Facebook and Instagram, and through posts in industry network pages on Facebook) and through leaders of the PT educations and leaders of the national fitness center chains in Norway. The survey was completely anonymous, and hence no personal identification such as work site, IP-address or contact information were collected. Inclusion criteria were Norwegian language skills at the B2 level, and operative in the PT profession at least during the four last weeks leading up to the Covid-19 pandemic lockdown. In total 172 PTs responded to recruitment, of which 150 fulfilled the inclusion criteria and completed the survey.

### Survey

The digital survey included demographic data, specially designed questions relating to the objectives in this survey (i.e., personal economic consequences of lockdown, and interpretation of regulations pertaining the Covid-19 lockdown, including initiation of new services), open-ended questions related to the closed questions, and the standardized instrument Subjective Vitality Scale (SVS).

### Demographic Data

The demographic data included gender, age, highest general- and exercise specific education, years of experience as PT, other work commitment, and work situation during Covid-19.

### Developed and Designed Survey Questions

The survey consisted of items and questions related to the lock down and experienced changes in working and living conditions. The project group strategically selected three PTs with differences in gender, education and employment status to give input on items and topics that were considered necessary to include in the survey. Written feedback from these PTs were then used for development of the questions described in Personal opinions and interpretations of regulation for exercise-services during Covid-19, Economic situation/consequences from Covid-19, Open-ended survey responses. The draft of the questions were sent to the PTs, and the final questions were revised following their comments and feedback.

#### Personal Opinions and Interpretations of Regulation for Exercise-Services During Covid-19

Eleven items were developed by the project group on (1) opinions to the lockdown of the fitness industry, (2) personal interpretation of the regulations, and (3) observed practice by colleagues, during Covid-19 lockdown. Responses were given on a 7-point Likert scale ranging from “strongly disagree” to “strongly agree” and a final option of “I don't know.” Examples of questions were: “PTs have interpreted the regulations differently” and “It feels like an unfair treatment of PTs and other operators within exercise-services with regards to the restrictions.”

#### Economic Situation/Consequences From Covid-19

We developed questions about other occupational work, whether the respondents were temporarily laid off from their PT occupation and/or other occupations, whether the lockdown affected their personal economy, whether they lost clients during the lockdown, if they believed the clients would return after the lockdown, and finally; whether they qualified for- and received financial support by the government. We also asked about any attempts to study for increased professional competence during the lockdown, and about their initiative to develop new PT services/new ways of delivering PT services (e.g., online training).

#### Open-Ended Survey Responses

The respondents were given the opportunity to formulate and express their opinion about eight areas related to the impact of Covid-19. These were about (i) economic compensation arrangement from The Norwegian Labor and Welfare Administration (NAV), (ii) the governmental guidelines and restrictions to control the transmission of the disease, (iii) new and innovative forms of exercise that PTs offered their clients, iv) feedback from the clients about their exercise habits before and after the outbreak of Covid-19 and the national lockdown, (v) the relations between the PTs and their clients, (vi) changes in these relationships due to lockdown, (vii) the PTs viewpoints regarding their health, exercise behavior and quality of life during the lockdown, and (viii) other comments about the future as a PT. These open-ended questions gave the respondents the possibility to freely express their experiences, feelings and emotions related to the effects of the pandemic, without limiting or influencing them with predefined answers. Asking open-ended questions may elicit surprising answers, new ideas, and unexpected experiences. Often such freely formulated answers are raw, unfiltered, and emotional. Many of them give colors to their experiences, as well as providing a deeper insight to the predefined questions they have just answered. These qualities were likely to be lost if we coded the answers according to our interpretations. In other words, we gave the respondents a voice beyond the statistics. In the following, the data from the open-ended questions are presented as an elaboration of the responses to the close-ended questions. However, not every respondent filled out the space in the open-ended questions. We therefore indicate how many respondents who used this option when presenting the open answers. These answers were not always very interesting, usable, or clear. Nevertheless, they are authentic and genuine answers in a frustrating, even frightening situation, and should be taken seriously in a project like this.

### Subjective Vitality Scale (SVS)

The SVS measures subjective vitality, i.e., the state of feeling alive and alert (Ryan and Frederick, 1997). It consists of seven items scored on a 7-point Likert scale (1, strongly disagree; 7, strongly agree), with optimal validity (Ryan and Frederick, 1997). One of the seven items is negatively worded, hence reversed scored for the analysis. The mean score was calculated with higher scoring indicating higher vitality. The respondents completed two versions of the SVS, one where they were asked to rate how they were normally feeling (i.e., before the Covid-19 lockdown) and one version where they were asked to rate how they felt the past 2 weeks before completing the survey (i.e., during the Covid-19 lockdown). Cronbach's alpha was 0.73 for the “before Covid-19 lockdown” -version and 0.76 for the “past 2 weeks” -version.

### Data Management

The data were obtained through the online survey tool Nettskjema (www.uio.no/nettskjema). The responses are anonymous, which means that information such as IP addresses were not collected/registered. According to the Norwegian Data Protection Services, we did not collect information that were potentially identifying, or sensitive data about the respondents, hence no approval for the study was needed. All data are held within the scientific group. Data sharing may be possible on request.

### Statistics

Data were analyzed by IBM SPSS Statistics version 26. All data were visually inspected for normality, and continuous data are presented as mean (SD) or median (range) as appropriate, while categorical data are presented as percentage. Differences between gender were analyzed by students' *t*-test or Mann Whitney *U*-test as appropriate for continuous data, and with Pearson's chi-square test for categorical data. A significance level of 0.05 was considered sufficient to discover any differences.

## Results

### Demographics

Half of the respondents reported general education from university/college on graduate or post-graduate level (Table 1), and 13% of the respondents had higher education with authorization as health personnel. A higher proportion of male vs. female respondents had a university degree in exercise science (Table 1). Nearly half of the respondents reported other paid work besides the work as a PT, with 14% working in health services and 10% in the teaching/educational sector. Of the 79 respondents with employment contracts as PT, 89% reported layoffs during the Covid-19 lockdown period. Of these, 49% reported resuming of the work as PT before reopening of the fitness centers at June 15th 2020.

**Table 1:** Descriptive data presented for male, female and total respondents.

	**Male (*n* = 57)**	**Female (*n* = 92)**	**Diff**	**Total (*n* = 150)^**a**^**
	**Mean (SD)**	**Mean (SD)**		**Mean (SD)**
Age, yrs	35.3 (8.4)	35.4 (8.2)	*t* = 0.09	35.5 (8.4)
Experience as PT, yrs	8.4 (6.0)	5.3 (4.5)	*t* = 3.57^***^	6.5 (5.3)
	n (%)	n (%)		n (%)
General education level, BA or MA	26 (46)	48 (52)	χ^2^(1) = 0.61	75 (50)
Exercise-specific higher education, BA or MA	22 (39)	19 (21)	χ^2^(1) = 5.68^*^	42 (28)
**Work as PT**				
Self-employed Contract with fitness center	36 (63) 23 (64)	48 (52) 29 (60)	*χ^2^* (1) = 1.73 χ^2^(1) = 0.11	84 (49) 52 (62)
Employed full time	18 (32)	39 (42)	χ^2^(1) = 1.74	57 (33)
Employed part time	6 (11)	15 (16)	χ^2^(1) = 0.97	22 (13)
Other work besides PT	29 (51)	42 (46)	χ^2^(1) = 0.39	72 (48)

a One respondent refused to report the biological sex and is therefore excluded from the comparative analyses between males and females. The respondent is included in the total population. PT: personal trainer. BA: bachelors degree. MA, masters degree; χ^2^(1), chi-square (df); t, t-value.

* p < 0.05.

*** *p < 0.001*.

### Operational Activity

**Figure 1:**
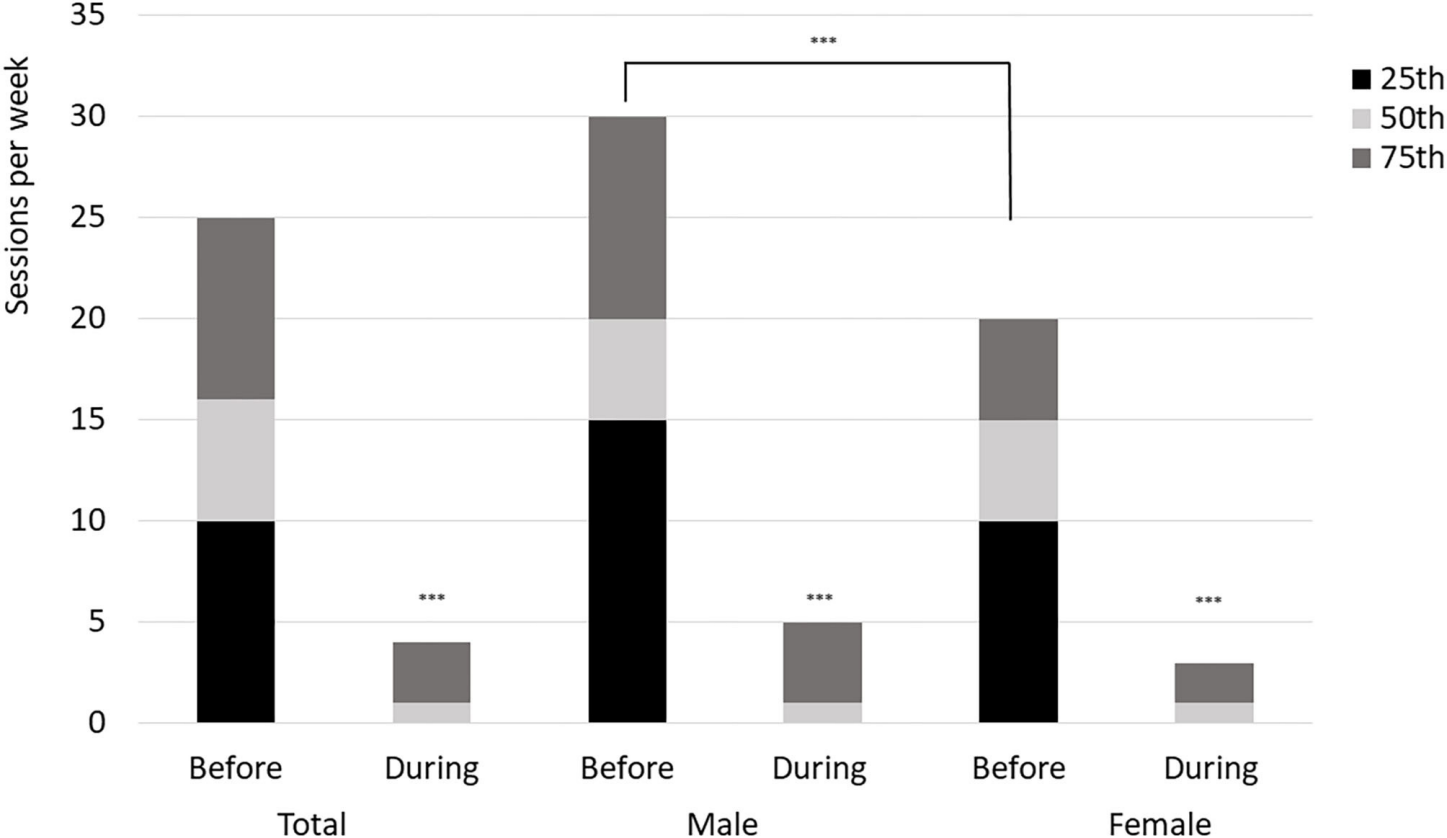
Number of personal training sessions per week before and during Covid-19 lockdown. ***difference between before and during (*p* < 0.001), and between males and females (*p* < 0.001).

Mean (SD) number of sessions per week before Covid-19 lockdown was 18.8 (14.1), with range from 2 to 100 sessions per week. Males had higher mean number of sessions per week prior to Covid-19 lockdown compared to females (Figure 1). During Covid-19 lockdown, number of sessions per week was reduced to 2.9 (5.3) with range from 0 to 40.

**Figure 2:**
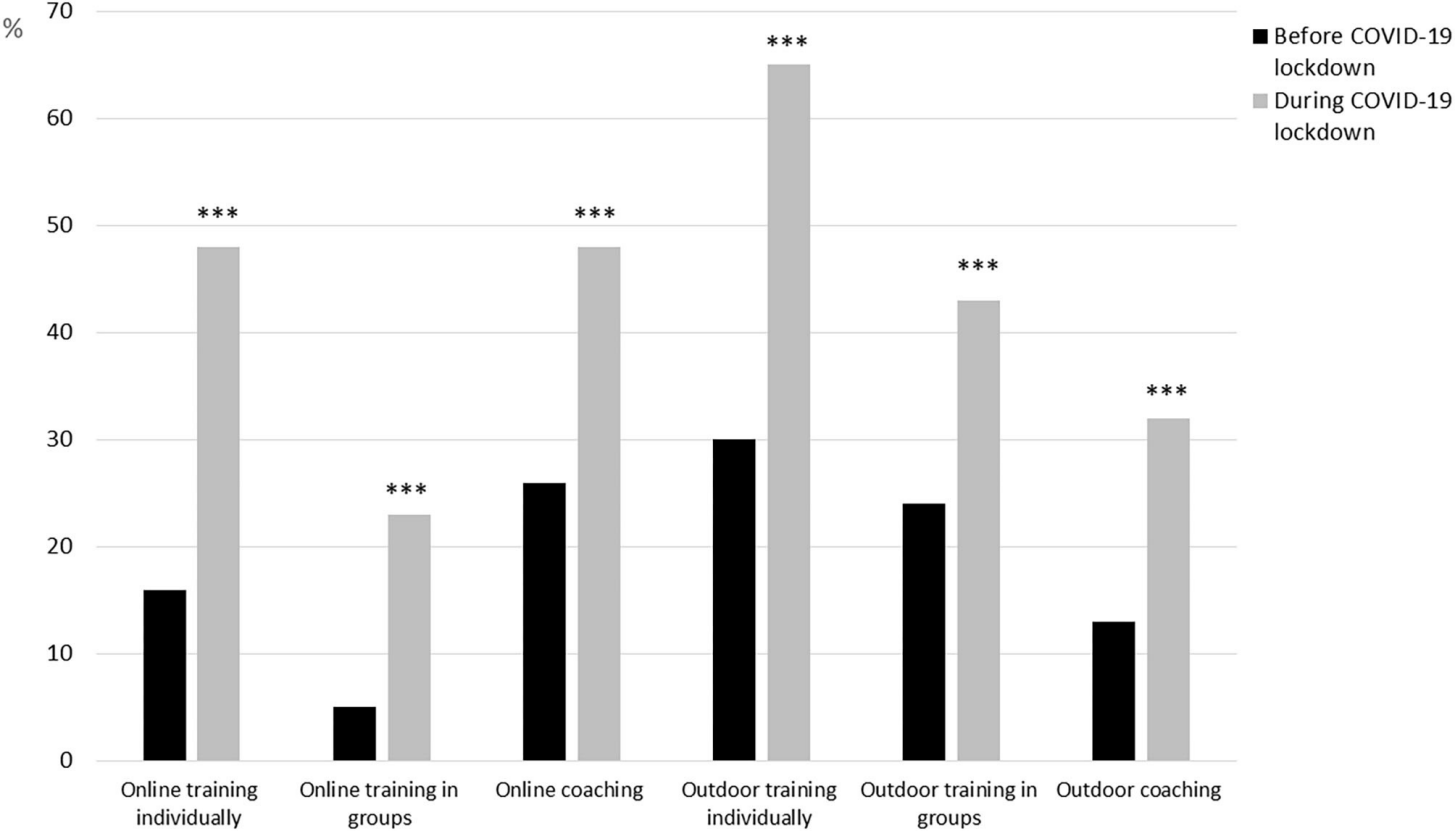
Personal trainers' provision of online and/or outdoor offers to clients before and during Covid-19 lockdown. ***p < 0.001.

Several of the respondents reported initiation of online and/or outdoor sessions for their clients during the Covid-19 lockdown, and online training was offered by three times as many PTs during Covid-19 lockdown compared to before the Covid-19 lockdown (Figure 2). A higher number of male respondents (58%) compared to female respondents (41%, *t* = 3.68, *p* = 0.03) reported use of online coaching during Covid-19. From the 31 commentaries about the new products, we learned that some moved the exercise outdoors keeping social distance. Others did online live-streams or established a Facebook group. However, most commentaries showed that these activities generated little income or led to expenses, as they often were free to clients and some of the online services such as *MyPTHub* meant extra costs to the PTs. The commentaries also showed that few clients wanted this service.

### Economic Situation and Governmental Compensation Offer

A total of 87% reported that the Covid-19 lockdown had affected their private economy, and 59% reported that they received financial support from the NAV. We asked the respondents to describe their own experiences with the *ad hoc* official compensation arrangement by NAV. Of the 150 participants in this study, 66 commented. The responses indicated great variations with NAV to perform homogeneous casework. Some expressed satisfaction with the arrangement. One respondent reported: “*This arrangement worked very well. Fast casework, smooth application procedure, and great services.”* Others complained about the arrangement and the processing time: “*[The arrangement works] … rather bad for me. Long processing time, no one at NAV can answer my questions, many contradictory answers.”* However, quite a few found the arrangement ok when it finally was up and running: “*It has worked well when finally activated. The solution was simple, and the money arrived fast.”* Importantly, economic compensation from NAV did not cover fully the ordinary income of the respondents. Many suffered from economic difficulties, which frustrated some of the respondents: “*We only received a symbolic amount of money to cover our overheads.”*

### Customer Base and Relations With Clients

Sixty-six percent of the respondents reported loss of clients due to the Covid-19 lockdown. Of these, 50% reported insecurity about, and 30% reported that they felt secure about, that the lost clients would return after the lockdown. Most of the 98 comments regarding clients' responses during the lockdown concerned a feeling of “privation” and “loss.” “*Miss gym - Miss me”* is a general comment in the open-ended question responses from the PTs. However, some of the PTs explicitly wrote that the clients approved the lockdown: “*Most of the clients recognized the situation but missed the opportunity to exercise at the center.”* The comments from several of the PTs also express a concern for the clients' reduced opportunity to exercise, their reduced motivation to exercise during the lockdown, yet their eagerness to start training again: “*Almost everyone have lost their motivation and have put their exercise on hold. Most of my clients express sadness about the closure of the center, but are looking very much forward to its reopening.”* Some PTs are also concerned about whether the clients will come back after the lockdown. They have registered that some clients are afraid of being infected: “*Based on an assumed insecure future, some of my clients do not want to spend money on PT. Many are afraid of a GREAT risk for infection.”*

A total of 88 respondents commented on impact of the lockdown on the relations to the clients, revealing a diversity of effects. Many indicated that the contact with the clients had suffered, with comments like “*less contact,” “I haven't seen them in months,” “I have to a certain degree lost the good relations we had built before the lockdown,” “Some have not answered my texts and emails,” “More distance between us”* and so forth. In addition, while one said: “*Some are easy to communicate with. Others are more difficult.”* other respondents stated: “*I have come closer to some of my clients. In a way, we have developed a new product together,” “I feel a more intimate and confident relation to some of my clients,” “The relation to old clients have grown stronger. The relation to new clients, are falling apart or has become non-existent.”* To some PTs, this bettering of relationships is attributed to being outdoor where the risk of being infected was reduced.

However, several of the PTs expressed concerns about their clients' motivation and drop out under the lockdown, but also after. “*Due to the insecure future, not everyone [of the clients] is willing to spend money on a PT. Many fears that exercise imply HIGH RISK for exposure to infection. Therefore, many have been very inactive.” “[Some of my clients] … are reluctant to start exercising again due to the Covid-19.”* Reading the commentaries, one gets the impression that the PTs are anxious about the industry's future. They express concerns about whether they will work as a PT in the future, and whether people will come back to the fitness centers. It seems that their concerns are rooted to their conviction that the PTs and the industry represent an important contribution to public health.

Both the quantitative data and the responses to the open-ended questions indicated that the PTs perceive the base of clients to be rather fragile and unpredictable with regards to future income. The intersection between privation, motivation, fear of Covid-19 upon return to exercise, communication and interaction with the clients, all tell a story about insecurity, uncertainty and concerns about the future. For the industry, this ought to raise some concerns about the ways commercial exercise are organized as well as the type of employment the PTs have in the organization.

### Perspectives on Rules and Regulations During the Covid-19 Lockdown

The majority agreed on the necessity of lockdown for the fitness center facilities from March 12th 2020, but due to the lack of specific guidelines for PTs, they reported difficulties in interpretation of operational opportunities (Table 2). They found the restrictions/guidelines unclear and difficult to understand and interpret compared to restrictions/guidelines for other exercise occupational groups (e.g., physiotherapists). There was a general perspective among the respondents about the restrictions/guidelines being discriminative between various professions and occupational groups (Table 2). A total of 40 respondents (27%) reported that they had completed a course in communicable disease and infection control, and 14 (8%) had completed such a course in their work as PT.

**Table 2:** The personal trainers' perspectives on the restrictions and guidelines during Covid-19 lockdown.

	**Disagree**	**Either/or**	**Agree**	**Don't know**
	***n*(%)**	***n*(%)**	***n*(%)**	***n*(%)**
The lockdown of fitness/training facilities was necessary	57 (38)	9 (6)	82 (55)	2 (1)
**The restrictions/guidelines applied to PTs during Covid-19 were…**				
…clear	83 (55)	15 (10)	51 (34)	1 (1)
…too strict	33 (22)	19 (13)	96 (64)	2 (1)
…easily understandable compared to restrictions/guidelines for other exercise occupational groups	95 (63)	20 (13)	31 (20)	4 (3)
I adhered to the guidelines provided for organized sports	25 (17)	26 (17)	91 (61)	8 (5)
I adhered to the guidelines provided for outdoor physical activity	5 (3)	12 (8)	128 (85)	5 (3)
**PTs have been…**				
…generally loyal to the restrictions/guidelines	21 (14)	12 (8)	96 (64)	21 (14)
…interpreting the guidelines differently/individually	14 (9)	17 (11)	104 (69)	15 (10)
…discriminated with regards to restrictions/guidelines that have been applied to other exercise occupational groups	12 (8)	13 (9)	123 (82)	2 (1)
Many PTs have not been loyal to the restrictions/guidelines	33 (22)	26 (17)	59 (40)	32 (21)

In the 29 commentaries to the open-ended questions about the restriction and guidelines, there was frustration about too absolute and oversimplified guidelines. Some respondents argued that there are important differences between large and smaller fitness centers with regards to exposure to infection. Others accused the guidelines to be unclear and that this had unintended consequences: “*Vague governmental communication early during the lockdown created great insecurity and disagreements in the industry.”* Some found it difficult to understand that a one-to-one session with a PT was hazardous considering the request for social distancing. In general, the respondents expressed an understanding for the lockdown during the 1st weeks, but this understanding vanished when other sectors and occupations, such as bars, restaurants and tattoo studios, were allowed to reopen when fitness centers were in lockdown until June 15th 2020: “*I support the lockdown until the 28*^*th*^ *of April and agree with the decision. However, I cannot comprehend that piercing and tattooing were allowed the 28*^*th*^*, but not to have indoor instructions from PTs.”*

### Self-Reported Health and Wellbeing

**Figure 3:**
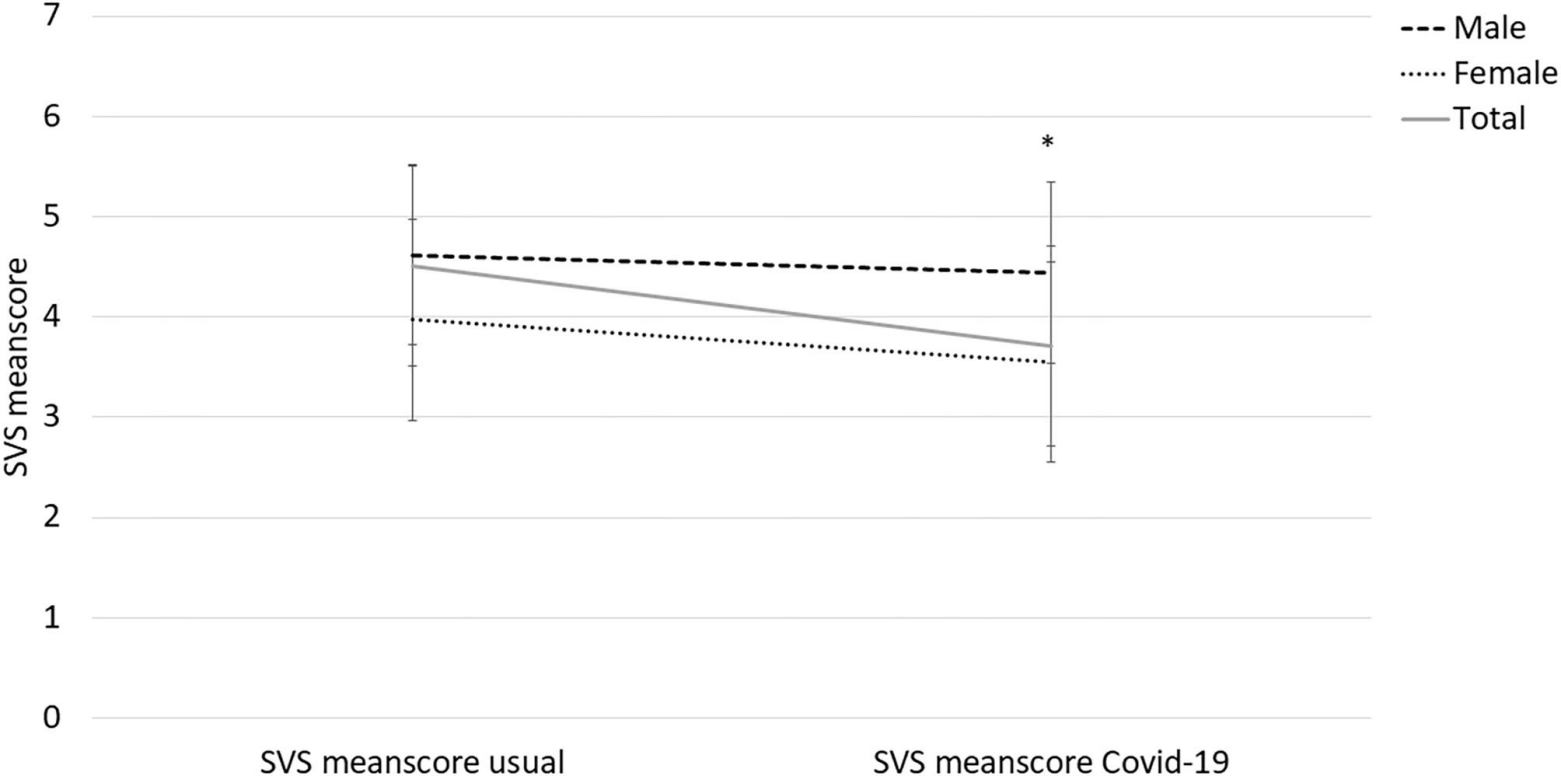
Changes in Subjective Vitality Scale (SVS) score among personal trainers before and during the Covid-19 lockdown period. *difference between males and females (*p* < 0.05).

**Figure 4:**
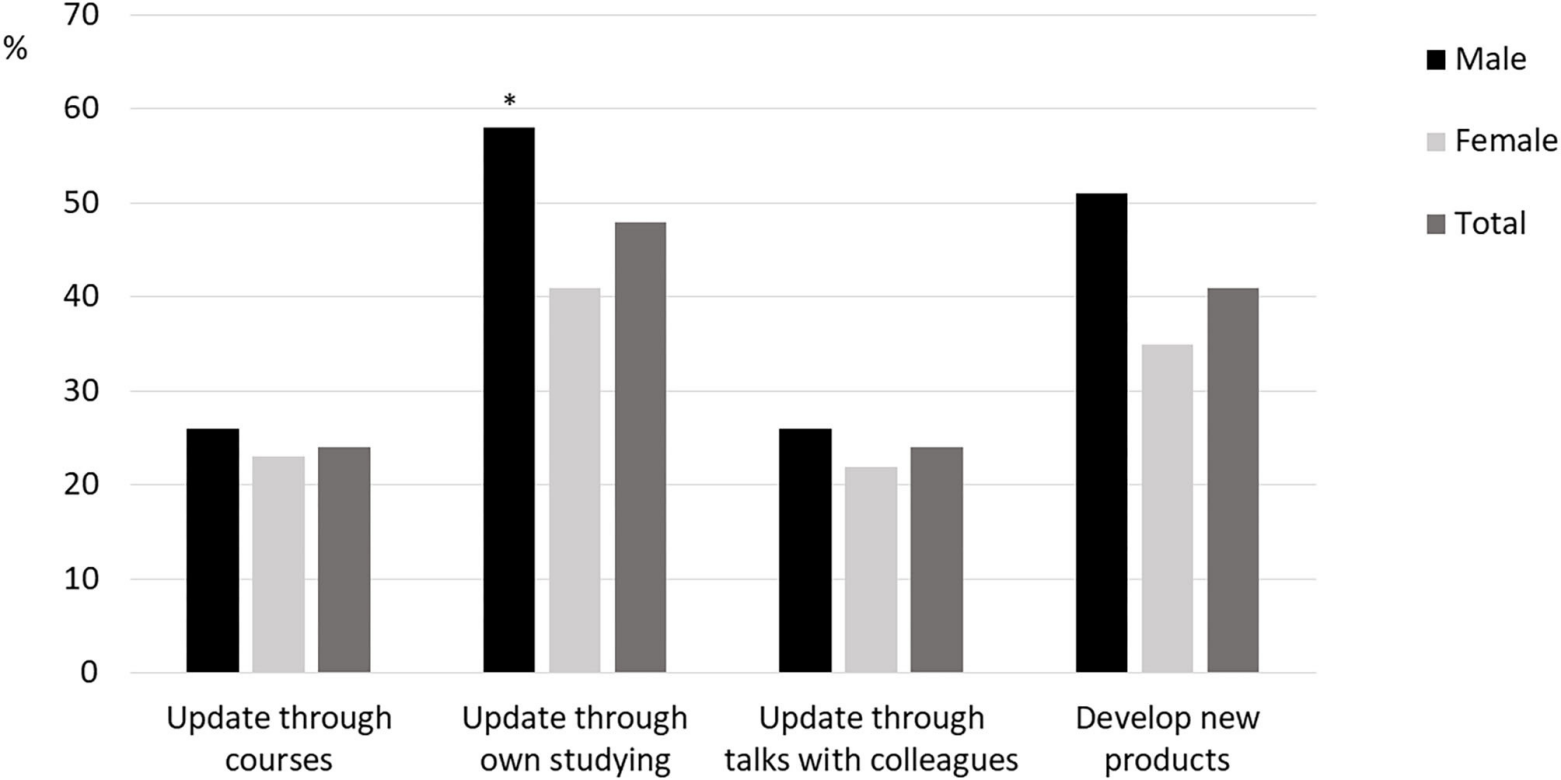
Personal trainers' use of the Covid-19 lockdown to other work-related activities. *difference between males and females (*p* < 0.05).

The respondents reported reduction in vitality (*p* < 0.001) from how they normally feel to how they felt during the Covid-19 lockdown. During the lockdown, females reported lower levels of vitality compared to males (3.55 vs. 4.44, Figure 3), while no gender differences existed for their usual SVS score. Sixty-two percent of the respondents reported that they managed to continue with their own physical activity and exercise behavior during the lockdown period. A higher percentage of the male compared to female respondents reported use of the Covid-19 lockdown period for update through own studying (*p* < 0.05, Figure 4).

Answers to the open-ended questions on the issues of own health, wellbeing and exercise were given by 48 PTs. The main impression is that Covid-19 had a detrimental impact on their health, quality of life, and exercise routines. Words used to describe the situation were often “*lowered energy-level,” “wear on the psyche,” “depressed,” “stressed,” “darkness,” “demanding,” “reduced motivation,” “lowered self-esteem,”* and so forth. For example: “*Both spirit, health and job satisfaction were low in this period.” “It wears down my psyche to be at home without a job. Not having something to do regularly, in addition to the uncertainty about income and how the employer takes care of us, is tiresome.” “… working 12–14 h a day to get a decent salary, [and now almost nothing] creates a great vacuum.”* Some missed their clients as well as their colleagues.

However, some reported positive changes during the lockdown. They had started to appreciate the job as an important social arena while others considered to study more or were actually studying during the lockdown period (see Figure 4). One respondent called for “*Leading by doing. It's of no use preaching about good health and quality of life through exercise and not follow up yourself. I'm taking my recommended medicine myself now.”* Quite a few experienced “ups and downs,” having spare time, time to think through the current situation, and the future. One comment sums up rather precisely most of the experiences revealed in the commentaries:

“*In the beginning, I was very depressed. I don't cope with being at home. I have lately started to appreciate more leisure time and time to rest. However, I have trouble maintaining sound routines for sleeping and eating. In sum, I am less active; eat more and less healthy… I am looking forward to coming back to daily routines, but wonder if ordinary days should be different from what they have been the last years. Despite challenges in this period, the circumstances have forced me to reconsider and rethink … [giving me]… time to deal with anxiety for having nothing to do.”*

### Thoughts About the Future as a Personal Trainer

Eighty-seven percent of the respondents considered it likely that they were still working as PTs in 6 months, and 21% were pessimistic about the future as PT. When asked about the PT occupation's role in public health work, 98% of the respondents reported that they considered their job as important for the public health. They considered this task as particularly important for the future work as PT. This is both strengthened and nuanced in the comments. Many believe that their contribution to public health has become clearer through the lockdown. One respondent said: “*I look forward to the day when I get acknowledged as a health worker.”* However, quite a few uttered concerns about factors that had to be clarified if this should become a reality. Eighty-two percent of the respondents reported the strong need for a union/organization for exercise professionals to secure a stable income – preferably a regular paycheck. Some also argue that online coaching and exercising outdoors likely will be more common in the future.

## Discussion

The aim of this study was to examine the impact of Covid-19 and the enforced lockdown on the work and living conditions of the PTs. Our main findings were that the lockdown had significantly negative, yet potentially unnecessary impact, on the working and living conditions for the PTs. We will in the discussion argue that the negative impact could have been avoided by more concise and specific regulations for the PTs from the start of the lockdown, and we will interpret this considering the known poor reputation of the fitness industry (Klepaker and Norøy, 2019).

### Gender Differences in the Reported Changes to Working and Living Conditions

With the lockdown, number of PT sessions per week was significantly reduced and many were either temporarily laid off by their employers or experienced sufficient loss in income to qualify for the *ad hoc* financial compensation from the Norwegian authorities/government. The lockdown period led to a reduction in self-reported vitality, especially among the female respondents. Simultaneously, there was an increase in use of online and/or outdoor training during the lockdown especially among the male respondents. Also, more males than females reported use of the lockdown period to study and/or follow courses. These gender differences can be compared to what have been reported in academia during the lockdown; finding fewer paper submissions and projects initiations among females compared to male researchers (Viglione, 2020).

Regarding the latter, one important reason for this gender difference was that female researchers to a greater extent than males had family obligations with home kindergarten and home school during the lockdown period. Family obligations have also been identified as the most plausible explanation for gender differences in sick absence from work in Swedish employees. We did not collect data on the respondents' family situation, and can therefore only speculate that inequality in family obligations might explain the detected gender differences. Future studies should test this hypothesis thoroughly. A second hypothesis for the observed gender differences has to do with masculinity and health information behavior (Courtenay, 2000; Ek, 2015). Previous studies show that men take less precaution and they care less about health information and behavior, and perhaps this form of masculinity became prominent with the pandemic and the lockdown. This can thus explain why the males kept the PT business going to a greater extend that the females.

Assuming PTs are vital and in good health, we were surprised to find lower SVS scores in our sample before the lockdown compared to what have been reported previously in university students, youth sport athletes and vocational dancers (Castillo et al., 2017). The female respondents in our study also showed lower scores on SVS compared to Norwegian early adolescents (Schmidt et al., 2020). One potential explanation might be that vitality is a unidimensional, dynamic construct of well-being sensitive to changes in e.g., life events (Ryan and Frederick, 1997), hence we cannot exclude the possibility that the retrospective “before Covid-19 lockdown period” has a limited validity in this sample due to completion of the instrument during the difficult times of the lockdown. The open-ended answers about health and wellbeing further indicated that the lockdown had detrimental effects on the PTs living conditions with reports of mental health challenges.

### Perspectives of the PTs Considering the Fitness Industry's Reputation

Our findings point to an initial approval and respect of the lockdown among the PTs, yet several questioned the necessity of keeping the fitness centers closed for 13.5 weeks as other health and wellness businesses such as tattoo studios, hair salons, spa and massage therapy and physiotherapy/chiropractic were allowed to re-open after ~6–8 weeks. The lack of union organization and lack of formal licensing with protection of the title “PT” have emerged specifically disadvantageous in a situation like the Covid-19. Our findings points toward frustration and a feeling of conflict as they identified themselves as public health providers, but were imposed restrictions and rules, which were difficult to interpret. Not being directly addressed in the official regulations during Covid-19, resulted in different interpretations of work opportunities among the PTs. The commentaries given in the open-ended questions in this survey, revealed a diversity in temporarily solutions among PTs. Many waited for the fitness facility to reopen, still others identified their service either in line with other health professionals or with organized sports and started private services when these were allowed to reopen their practices. Some PTs even found their operative activity reasonable as long as they adhered to the public regulations (i.e., operating in small groups, keeping a social distancing of 2 meters) (Bugge, 2020). This diversity in interpretation resulted in an impression that some PTs were not being loyal to the guidelines, which can be a source for conflict. What seems to be the core of the difference in interpretation of the guidelines is the outdoor training. This potential discord and conflict among the PTs could have been solved by more explicit guidelines for organized outdoor training from the government. As mentioned in the Introduction, *Virke trening* provided several attempts to develop such guidelines and have these reviewed and approved by the government (Kristiansen, 2020a), yet the Norwegian health authorities were reluctant to do so. One reason for why the authorities overlooked the PTs in their specific guidelines and priorities when reopening the community, may be a reputational history of the fitness industry as a body appearance focused arena, with less health related motivation (Klepaker and Norøy, 2019; Kristiansen, 2020b). Hence, when health authorities prioritized the first services to reopen, the PTs and fitness industry was not considered sufficiently important prior to other health and wellness services. Other than being overlooked as a health promoting service, the activities typically associated with the fitness industry could also cause concerns about infection control. One suggested explanation for the reluctance to reopen the fitness center facilities by the government was that the typical fitness center activities takes place inside crowded facilities using shared equipment, and that services by PTs are associated to these arenas (Kristiansen, 2020b). Previous studies have also shown poor sanitation of surfaces in fitness centers (Mukherjee et al., 2014; Maurice Bilung et al., 2018), and it is plausible to believe that the decision-makers therefore were unsure about the industry's ability to adhere to strict guidelines and regimes for sanitation during a pandemic.

Additionally, with the encouragement of authorities and the allowance of using outdoor, recreational physical activities during the lockdown (Seljeseth, 2020; The Norwegian Directorate of Health, 2020), the decision-makers might have had an impression that the general public did not need the fitness center facilities in order to stay physically active during the pandemic. Given the popularity of outdoor physical activities in Norway, and the image of Norwegians as an “outdoor and hiking people” (Gurholt and Broch, 2019), this was a reasonable assumption. However, the decision-makers seem to have neglected the empirical knowledge that more than 30% of the Norwegian population exercise regularly in fitness centers (Breivik, 2013; Virke Trening, 2019). Despite well-known social inequalities in health and physical activity level, outdoor life is the only arena for physical activity where the effect of social class seem to be negligible (Breivik, 2013), and hence it can be argued that the highest social classes were most affected by the lockdown of the fitness centers. The picture is however more complex, as those in high social class are often more motivated to exercise by themselves and for health related reasons compared to the lower social classes (Breivik and Rafoss, 2017). Some of the open-ended comments by the respondents also points to the social connection enabled by PTs in both individual and group exercise. The fitness centers' role in social life and how their lockdown for 13.5 weeks affected the clients' social network and isolation should therefore be examined thoroughly in future studies. Also, more knowledge is therefore needed on how social inequality in health and physical activity might have been impacted by the lockdown of fitness centers.

The PTs reveal an apparently genuine concern about the health and wellbeing of their clients during the lockdown period, and an understanding and insight into the complexity of exercise behavior. This finding must be viewed in light of the relatively high educational level among the PTs who responded to our survey, but nevertheless this insight is in our opinion an argument for the PTs general level of competence and thus that they are under-evaluated as public health ambassadors. We also find that the concerns about the loss of clients not necessarily was related to their own loss of income but also an expression of idealism, which is in line with a previous finding (Rahman and Wills, 2013). The use of more or less free online and/or outdoor offers for the clients also add up to this interpretation. The restrictions for fitness centers were perceived as oversimplified and can add up to the impression that the government is not sufficiently familiar with the diversity of fitness centers and the work of PTs.

### Covid-19 Lockdown as a Window of Opportunity?

Based on the findings of our study, there is reason to say that the Covid-19 lockdown has illuminated several issues for the PTs as a profession and for the fitness industry as discussed in Perspectives of the PTs considering the fitness industry's reputation. This can also be viewed as a window of opportunity for discussion and change both internally in the industry, but also toward stakeholders and decision makers within public health. On organizational level, we argue that this lockdown period, the experiences and how it was handled, can serve as an opportunity for the fitness industry and the profession of PTs to unite and demand changes. For instance, development and implementation of an accreditation system will likely improve the reputation and how the PTs' competence is viewed and acknowledged, and further improve professionalism of the industry.

Despite the identified negative impacts of the Covid-19 lockdown period, the majority of the respondents were positive about the future as a PT. They rate their own services as important public health work, and they find their profession needed also in the years to come. Perhaps the Covid-19 will affect the public health negatively both on short and long term, and such an effect will require much effort and collaboration between sectors and professions. Moving services into online platforms, outdoors, and development of new products provides other possibilities and potentially new groups of clients for the PTs. Although it was reported that moving services online during the lockdown generated little income, and actually provided economic loss for some PTs, this must be seen in relation to the rapid change of political responses to the pandemic and that the lockdown happened over night. Such digital services might have a potential for generating income when this can be planned and developed properly by the PTs. Nevertheless, the importance of social connection and meeting clients and colleagues face to face should also be addressed in future studies concerning PTs working conditions and occupational health. Our data show the genuine care and concern PTs show for their clients, and how this client/PT relationship can be kept and further developed is an area for future research.

### Implications of the Findings

Practically, the findings call for more concrete and specific guidelines for how the work of PTs can be performed during pandemic situations. Such guidelines can be in accordance with the guidelines for other health and exercise professions. Also, the findings implicate the need for establishing a union organization for PTs, an organization that can take care of the needs of the PTs both toward their employers (i.e., large fitness center companies) and toward the government and health authorities. The findings, also documented in other studies, suggest a discussion of the PTs' educational level. Increased scientific knowledge, acquired through university degrees, may help the PTs to be more qualified and more accepted as health providers in the society. This will benefit the PTs, the industry and the society in general. In the aftermath of the pandemic, this should be a lesson learned, in this industry as well as in other industries within the field of culture.

### Suggestions for Future Research

The scientific implications of our findings are the need for follow-up on the working and living conditions of PTs on a more long-term basis. The descriptive nature of this study is suited to create hypotheses based on the findings. Based on the results from this study, we argue that the documented gender differences and the low scores on vitality calls for future research.

First, two factors that has been found important in explaining gender differences in work life and health are differences in family obligation (Angelov et al., 2013) and masculinity in health behavior (Courtenay, 2000; Ek, 2015). One important reason for the observed gender difference in Viglione (2020) was that female researchers to a greater extent than males had family obligations with home kindergarten and home school during the lockdown period. Family obligations have also been identified as the most plausible explanation for gender differences in sick absence from work in Swedish employees (Angelov et al., 2013). We did not collect data on the respondents' family situation, and future studies therefore need to collect this information to explore potential family obligation inequality among PTs. The importance of masculinity in health behavior manifests with men taking less precaution and care less about health information and behavior (Courtenay, 2000; Ek, 2015). Future studies should examine if this form of masculinity became more prominent with the pandemic and the lockdown, and if this can explain why males kept the PT business going to a greater extent than the females.

Second, the low scores on vitality combined with the reported negative impact of the lockdown on the working conditions calls for more research on the PTs general health, including somatic, mental, social and occupational health. Their motivation for becoming PTs and how this motivation affects their job motivation and job satisfaction should also be addressed in future studies. This motivation is important to understand because it might relate to their health. For instance, previously published anecdotes describe PTs who chose this profession due to themselves being helped to e.g., recover from illness and/or injury, improve performance, or that they somehow discovered the greatness of physical activity and exercise and then want others to have the same positive experiences (Howliston, 2016; Green, 2018). A study on health trainers' professional journey in UK (Rahman and Wills, 2013) showed that the health trainers were highly motivated by the value of “giving back” to their community, and this might also be an important motivation for PTs.

### Strengths and Limitations

This is to our knowledge the first study examining the working and living conditions of PTs as a profession. The study is limited by the self-report, the cross-sectional and retrospective design, and the recruitment through social media. The SVS was completed twice in the survey, one with focus on vitality prior to the lockdown and one version with focus on vitality during the lockdown. As they were completed during the same session, but with one version retrospectively, we must take into account that they might have over-evaluated how their vitality level was prior to the lockdown. Further, almost 30% of the respondents reported exercise-specific education from university/college on graduate or post-graduate level, this was higher than expected when comparing to a survey about the formal exercise-specific competence of PTs from 2016 (Virke Trening, 2016). Hence, the results may not be generalizable for the entire population of PTs. Although this survey has a quantitative approach, we allowed the respondents to comment to open-ended questions. These comments and answers were used as a supplement to elaborate on the quantitative findings, and they were voluntary to complete. Hence, not all responded to these open-ended questions and the content of the comments varied from one-word phrases to longer narratives. We acknowledge that this limits the possibility for a deeper and more qualitative understanding of the PTs experiences and perspectives.

## Conclusion

This study showed that the working and living conditions of Norwegian PTs were detrimentally affected by the Covid-19 lockdown period. Some gender differences were detected, with females reporting lager reduction in working hours and in subjective vitality than males. The PTs acknowledged the need for lockdown and restrictions; however, they called for concrete and specific guidelines for their profession. Although the majority of the respondents reported continuance of own physical activity and exercise during the lockdown, almost 40% were not able to do so. The majority was also optimistic about the future of their occupation, yet we recommend that future studies follow-up on these topics among the PTs.

## Data Availability Statement

The raw data supporting the conclusions of this article will be made available by the authors, without undue reservation.

## Ethics Statement

Ethical review and approval was not required for the study on human participants in accordance with the local legislation and institutional requirements. The patients/participants provided their written informed consent to participate in this study.

## Author Contributions

SB-S had the idea to the project and did the statistical analysis. SB-S, TM, CS-B, JS-B, and JT developed the survey, performed the data collection, and wrote the manuscript. JT had responsibility for the commentaries on the open-ended questions. All have read and approved the final version of the manuscript.

## Conflict of Interest

The authors declare that the research was conducted in the absence of any commercial or financial relationships that could be construed as a potential conflict of interest.
